# Occupational Risks and Pregnancy and Infant Health Outcomes in Florida Farmworkers

**DOI:** 10.3390/ijerph110807820

**Published:** 2014-08-06

**Authors:** Jennifer Runkle, Joan Flocks, Jeannie Economos, J. Antonio Tovar-Aguilar, Linda McCauley

**Affiliations:** 1 Nell Hodgson Woodruff School of Nursing, Emory University, Atlanta, GA 30322, USA; E-Mail: davisjr6@gmail.com; 2 Center for Governmental Responsibility, Levin College of Law, University of Florida, Gainesville, FL 32611, USA; E-Mail: flocks@law.ufl.edu; 3 Farmworker Association of Florida, Apopka, FL 32703, USA; E-Mails: farmworkerassoc@aol.com (J.E.); tonytovar@hotmail.com (J.A.T.-A.)

**Keywords:** community-based participatory research, agricultural, occupational and environmental exposures, maternal and child health outcomes, female farmworkers

## Abstract

The agricultural industry has some of the highest incidence rates and numbers of occupational injuries and illnesses in the United States. Injuries and illnesses in agriculture result from accidents, falls, excessive heat, repetitive motion and adverse pesticide exposure. Women working in agriculture are exposed to the same hazards and risks as their male counterparts, but can face additional adverse impacts on their reproductive health. Yet, few occupational risk assessment studies have considered the reproductive health of female farmworkers. The objective of this community-based participatory research study was to conduct a retrospective, cross-sectional survey to collect information on workplace conditions and behaviors and maternal, pregnancy and infant health outcomes among a sample of female nursery and fernery farmworkers in Central Florida. Survey results showed that nursery workers were more likely to report health symptoms during their pregnancy than fernery workers. We also observed a self-reported increased risk of respiratory illness in the first year of life for infants whose mothers worked in ferneries. Our findings confirm that agricultural work presents potential reproductive hazards for women of childbearing age.

## 1. Introduction

Agricultural work is one of the most hazardous of all occupations. Limited research has demonstrated an association between certain agricultural tasks and adverse health outcomes in women and infants. For example, handling or being exposed to pesticides has been linked to reduced fertility [1,2,3,4,5], elevated breast cancer risks [6] and higher risks for low birth weight [7], birth defects [8,9] and infant mortality [10]. Relatively few studies have assessed the health of female farmworkers distinctly from their male counterparts or the impact of agricultural work-related tasks on pregnancy outcomes. Besides pesticide exposure, other occupational risk factors that are of concern for pregnant women include ergonomic tasks involving repetitive motion, heavy lifting, frequent bending and prolonged standing and prolonged work in high temperatures with limited access to drinking water [11,12,13,14,15,16].

In the past two decades, researchers have used community-based participatory research (CBPR) as a method of building trust with the farmworker community and gaining access to study the health risks in this vulnerable population. This paper presents the cross-sectional survey results of a CBPR study on the occupational and reproductive health risk factors for farmworker women who worked during pregnancy and the prevalence of self-reported pregnancy, birth and infant health outcomes in this population.

## 2. Materials and Methods

This CBPR project was a collaboration between academic researchers from Emory University (Emory) and the University of Florida and the community-based Farmworker Association of Florida (FWAF) formed to investigate the impact of occupational and environmental exposures on the health of female farmworkers and their infants. The FWAF is a grassroots farmworker organization with a 30-year history in the community and with former farmworkers as staff members and a board of directors of mostly farmworkers. In the environmental health arena, CBPR provides the means to address the procedural, geographical and social inequities that communities may experience by engaging community members in problem definition, study design, data collection and interpretation and policy applications [17]. The community identified the problem to be studied, which was the concern that women working in agricultural are potentially exposed to chemicals and other hazards that could affect their pregnancy health. Members of the FWAF served on the research team, and workers who lived in the community were trained to participate in data collection, including recruiting and interviewing women who participated in the study.

### 2.1. Participants

In the summer of 2011, we recruited 260 farmworker women ages 18 to 40 who had been working in Central Florida nurseries and ferneries for at least six months and who worked at least 20 h a week in agriculture. We relied on snowball sampling for recruitment at farmworker housing, local churches, schools, area clinics, local ethnic businesses, community events, FWAF programs and other locations frequented by female nursery and fernery workers. Snowball sampling has often been used alone or in combination with other methods to recruit from hard to reach populations, especially in community-based research [18,19,20]. The majority of interviews were conducted at the FWAF offices in Pierson and Apopka, though some were conducted in the workers’ homes, in the late afternoons after work or on rare occasions during the weekend when workers were off. The participants provided information on current and past work conditions and their pregnancy histories. Participants received compensation of $25 and a “*Your Work and Your Health*” informational pamphlet developed by the FWAF to help farmworkers recognize signs and symptoms of pesticide exposure and to engage in protective measures. All study procedures were reviewed and approved by the IRB of Emory University, and consents and questionnaires were administered in English, Spanish or Haitian Creole, depending on the interviewee’s stated preference.

### 2.2. Setting

Farmworker participants were recruited from two geographic locations in Central Florida: (1) nurseries in Apopka, FL (Orange and Lake counties), an area frequently referred to as the “Indoor Foliage Capital of the World”; and (2) Pierson, FL (Volusia and Putnam counties), an area commonly referred to as “The Fern Capital of the World.” The FWAF has offices in both Pierson and Apopka, where community members come for services, referrals, assistance, membership meetings and events.

For this study, “nurseries” were defined as those companies producing a variety of ornamental plants, including cut flowers, flowering potted plants, hanging baskets, potted foliage, bedding and garden plants and woody ornamentals. Most nursery workers labor inside greenhouse structures enclosed with non-porous heavy plastic. Work tasks at nurseries are varied and may include: planting at conveyor belts; loading pots of plants onto trays; and loading and carrying trays, boxes or bags of soil. According to community-based estimates, there are 10,000 to 12,000 workers in the Central Florida nursery/foliage industry. Nursery workers are primarily of Mexican origin, but there are also indigenous people from Central America, Hispanics from other countries, Haitians and African-Americans. A previous study with Florida nursery worker households showed that a majority of workers (71%) were married or cohabiting and about half (50.2%) had children in the household [21].

“Ferneries” were defined as companies producing cut ornamental foliage, such as that used as floral greenery. Fernery workers labor in fields enclosed on all sides and across the top by porous black saran shade cloth or under tree canopy. To harvest ferns, workers bend over, thrust their arms into masses of ferns, cut fronds at their base, and secure them into bunches of 20 to 25 fronds. They leave the fern bunches on the ground until they have a particular quantity, then they gather up all of the bunches in their arms and quickly carry them to a trailer waiting at the edge of the field. The most experienced fern cutters can harvest up to 300 bunches of leatherleaf ferns a day. According to community-based estimates, there are an estimated 13,000 workers in the Central Florida fern industry. The majority of fernery workers are of Mexican origin, but there are also indigenous people from Central America. A previous study found that the majority of fernery workers surveyed (76.2%) were married or cohabiting and a large proportion (64.9%) had children in the household [21].

### 2.3. Study Instrument

A comprehensive cross-sectional survey was developed by the project team partners with input from a community advisory team and administered by trained community interviewers. The questions were developed using data from focus groups with women working in agriculture completed in the initial phases of this CBPR project [22,23]. Survey items were included to capture workplace conditions, as well as perceptions of workplace risks, health symptoms experienced by the women (relating to heat, ergonomic and pesticide workplace stressors) and pregnancy histories [21]. The survey obtained information on demographics (e.g., age, country of birth, native language and pregnancy status), work practices (e.g., work tasks, health symptoms), work-related hygiene (e.g., access to hand-washing facilities, availability of clean water, use of bathroom), health beliefs (e.g., perceptions of pesticide exposures and health risks), a brief lifetime pregnancy history and a detailed history of the most recent pregnancy (see the Supplemental Material for the survey). Study questionnaires were translated into Spanish and Creole by bilingual FWAF staff members who understood the meanings of the original questions and back translated by independent parties. Survey questions were pilot tested on a sample of 15 FWAF community members (*i.e.*, farmworker members) who previously worked in nursery or fernery operations. This article focuses on findings related to a woman’s most recent pregnancy history.

Pregnancy health-related categories included in this retrospective, cross-sectional analysis were: (1) the duration of work in agriculture while pregnant; (2) access to prenatal care and physician diagnosed health conditions (mother and child); (3) health symptoms during pregnancy; (4) pregnancy outcomes and infant health for the first year of life; (5) breastfeeding practices; (6) fathers work in agriculture; and (7) body pain discomfort. Paternal work in agriculture was determined by the mother reporting whether or not the father worked in agriculture during her last pregnancy. Women who reported experiencing musculoskeletal discomfort while working in agriculture during their most recent pregnancy were asked to complete a body pain discomfort chart. Musculoskeletal pain was categorized into the following nine areas: (1) neck; (2) chest and shoulder; (3) upper back; (4) mid-lower back; (5) upper abdomen; (6) lower abdomen; (7) upper arms; (8) lower arms; and (9) lower extremity.

*Pregnancy Outcomes.* For this analysis, we defined a pregnancy complication as one of the following events: miscarriage, abortion, stillbirth (>20 weeks) or ectopic or molar pregnancy. The two primary birth outcomes of interest were: (1) preterm birth (birth at a gestational age greater than 20 weeks, but less than 37 weeks); and (2) low birth weight (LBW) (infant delivered weighing less than 2,500 grams).

*Infant Outcomes*. Women were asked if their child had ever been diagnosed by a healthcare provider with any birth defects or health complications from the time of delivery until 1 year of age; responses were recorded as infant health complications (yes/no). Women were also asked if a healthcare provider had ever diagnosed their child with any health problems, including respiratory or breathing problems, psychological or learning problems and/or other chronic health problems in the first year of life. For this question, women were also asked to specify the diagnosis. Given that the majority of self-reported child health problems were respiratory-related (23 out of 30 responses), we created a bivariate respiratory condition outcome (yes/no). The two infant outcomes considered in the analysis were: (1) child diagnosis of chronic condition (yes/no); and (2) child diagnosis of respiratory condition (yes/no).

### 2.4. Statistical Analysis

Descriptive statistics examined differences in demographic, work-related changes while pregnant, musculoskeletal discomfort and maternal and infant health outcome characteristics between nursery and fernery workers using a Pearson chi-square or Fisher exact test for categorical variables and Student’s *t*-tests for continuous outcomes. A significance level of *p* = 0.05 was used, and all bivariate statistical analyses were performed using SAS 9.3 (Cary, NC, USA).

## 3. Results

A total of 260, 18- to 40-year-old, female Hispanic and Haitian nursery and fernery workers completed the survey. Data presented here are from 170 women (*n* = 62 nursery and *n* = 108 fernery workers) who worked in agriculture during their last pregnancy, were not currently pregnant (*n* = 22 excluded) and who provided information on their most recent pregnancy in the Pregnancy Historyportion of the survey.

While women from both agricultural groups were similar in age, these groups differed in important ways. A larger proportion of nursery workers reported being single or divorced (*n* = 22%, 35.6%), compared to fernery workers (*n* = 20%, 18.5%) (*p* = 0.03). While all fernery workers were Spanish-speaking and Hispanic, nearly one-third of nursery workers (*n* = 20) spoke Creole as their native language (*p* < 0.0001) and did not identify as Hispanic (*p* < 0.0001). On average, female fernery workers worked in agriculture longer than their nursery worker counterparts (10.7 years *vs.* 7.1 years, respectively) (*p* < 0.0001) and had two or more children (92.6% *vs*. 72.6%) (*p* < 0.001). Though not statistically significant, the majority of fernery workers (76.9%) reported living with the infant’s father during their most recent pregnancy compared to nursery workers (74.2%) (*p* = 0.08). The self-report of health risk behaviors during pregnancy (smoking and drinking) was very low in both groups of women. More women working in fernery operations (76.9%) reported that their child’s father worked in agriculture compared to women in nurseries (16.7%) (*p* < 0.001) (Table 1).

**Table 1:** Demographic characteristics of the Central Florida female nursery and fernery workers (*n* = 170), 2011.

Variables	Nursery (N = 62) *n* (%)	Fernery (N = 108) *n* (%)	*p-*Value
*Age (mean ± SD)*	31.9 ± 6.6	32.2 ± 5.7	0.78
*Years worked in agriculture (mean ± SD)*	7.1 ± 5.0	10.7 ± 5.2	<0.0001
*Work in agriculture during pregnancy*			<0.0001
Yes, in a nursery	59 (95.2)	6 (5.6)	
Yes, in a fernery	2 (3.2)	96 (88.9)	
Yes, both	0	3 (2.8)	
Yes, other agriculture	1 (1.6)	3 (2.8)	
*Marital status*			0.03
Married	28 (45.2)	57 (52.8)	
Living as married	12 (19.4)	31 (18.2)	
Single	14 (22.6)	17 (15.7)	
Divorced	8 (12.9)	3 (2.8)	
*Native language*			<0.0001
Spanish	39 (62.9)	108 (100)	
Creole	20 (32.3)	0	
*Hispanic*			<0.0001
Yes	42 (67.7)	108 (100)	
No	20 (32.3)	0	
*Nationality*			<0.0001
United States	0 (0)	4 (3.7)	
Mexico	34 (55.7)	104 (96.3)	
Haiti	20 (32.8)	0 (0)	
Other	7 (11.5)	0 (0)	
*Currently Employed ^a^*			<0.0001
Part-time (<35 h)	25 (40.3)	75 (69.4)	
Full-time (>35 h)	37 (59.7)	29 (26.9)	
*Years of Education completed in native country (mean ± SD)*	7.7 ± 4.0	6.6 ± 3.11	0.05
*Parity*			<0.001
1	17 (27.4)	8 (7.4)	
≥2	45 (72.6)	100 (92.6)	
*Smoke during pregnancy*			0.17
Yes	1 (1.6)	0	
Sometimes	1 (1.6)	0	
No	60 (96.8)	108 (100)	
*Drink during pregnancy*			0.19
Yes	0	0	
Sometimes	1 (1.6)	0	
No	61 (98.4)	108 (100)	
*Live with father during last pregnancy*			0.08
Yes	46 (74.2)	91 (85.1)	
No	26 (25.8)	16 (14.9)	
Did Father work in agriculture during last pregnancy? *			<0.001
Yes	17 (27.9)	83 (76.9)	
No	42 (68.9)	16 (14.8)	
Refused to Answer	0	1 (0.83)	
Don’t know	1 (3.2)	8 (7.4)	
Did Father apply pesticides at work during last pregnancy? *			0.28
Yes	10 (16.7)	11 (10.3)	
No	47 (78.3)	86 (80.4)	
Refused to Answer	0	1 (0.93)	
Don’t know	3 (5.0)	9 (8.4)	

^a^ Four (3.7%) fernery workers reported currently being unemployed; * only compares yes *vs.* no responses between each agricultural group.

### 3.1. Work-Related Behaviors during Pregnancy

Compared to nursery workers (88.7%), fernery workers (65.7%) were less likely to report being pregnant to their supervisors (*p* < 0.001), and when female fernery workers did tell their supervisors, they waited almost a month longer than nursery workers (*p* = 0.02). More than 97% of the women reported receiving prenatal care. Nursery workers reported entering prenatal care at 11.5 weeks and fernery workers at 11.6 weeks (*p* = 0.96). Fernery workers were more likely to be asked by a healthcare provider (HCP) to alter their work routine (*p* < 0.001) and were more likely than nursery workers to adhere to physician-suggested work alterations by decreasing their work hours (*p* = 0.02) and taking more frequent breaks (*p* < 0.001). Nursery workers reported sitting more than standing at the workplace (*p* = 0.02) following health provider suggested behavior changes at work (Table 2).

**Table 2:** Self-reported work-related behaviors while pregnant for female nursery and fernery workers in Central Florida (*n* = 170), 2011. HCP, healthcare provider.

Work-related behaviors	Nursery (N = 62) *n* (%)	Fernery (N = 108) *n* (%)	*p-*Value
*Worked during pregnancy*			0.86
Yes, entire	33 (53.2)	56 (51.8)	
Yes, partial	29 (46.8)	52 (48.2)	
*Week stopped working*	23.6 ± 8.1	22.0 ± 7.6	0.59
*Told boss about pregnancy*			<0.01
Yes	55 (88.7)	71 (65.7)	
Never told	4 (6.5)	19 (17.6)	
No	3 (4.5)	18 (16.7)	
**Prenatal practices**	**Nursery (N = 62) *n* (%)**	**Fernery (N = 108) *n* (%)**	***p-*Value**
*Received prenatal care*			0.54
Yes	60 (96.8)	105 (97.2)	
No	2 (3.2)	3 (2.8)	
Week entered pre-natal care			0.96
*(mean ± SD)*	11.5 ± 13.7	11.6 ± 6.3	
Asked to make work changes by HCP *			<0.001
Yes	20 (43.4)	54 (75.0)	
No	26 (56.5)	18 (25.0)	
Asked by HCP to cut hours *			0.02
Yes	4 (11.8)	18 (33.3)	
No	30 (88.2)	36 (66.7)	
Asked by HCP to take frequent breaks *			<0.001
Yes	7 (20.6)	33 (61.1)	
No	27 (79.4)	21 (38.9)	
Asked by HCP, to sit, instead of stand *			0.02
Yes	4 (11.8)	0	
No	30 (88.2)	54 (100)	

* Twenty eight nursery and 54 fernery workers did not provide responses to these questions.

Women working in ferneries while pregnant reported significantly more body pain discomfort in seven out of nine body sites, including the neck (34.3% *vs*. 10.5%) (*p* < 0.001), upper back (31.5% *vs*. 3.5%) (*p* < 0.0001) and mid-lower back (53.7% *vs.* 19.3%) (*p* < 0.0001), upper abdomen (40.0% *vs.* 15.8%) (*p* <0.01), lower abdomen (53.7% *vs.* 24.6%) (*p* < 0.001), lower arms (22.2% *vs.* 5.3%) (*p* < 0.01) and lower extremity (56.5% *vs.* 15.8%) (*p* < 0.0001) compared to nursery workers (Table 3).

**Table 3:** Self-reported body pain discomfort for nursery and fernery workers who worked during their last pregnancy (*n* = 170), 2011.

Body Pain Discomfort	Nursery * (N = 62) *n* (%)	Fernery (N = 108) *n* (%)	*p-*Value
*Neck*			<0.001
Yes	6 (10.5)	37 (34.3)	
No	51 (89.5)	71 (65.7)	
*Chest and shoulder*			0.05
Yes	6 (10.5)	25 (23.2)	
No	51 (89.5)	83 (76.8)	
*Upper back*			<0.0001
Yes	2 (3.5)	34 (31.5)	
No	55 (96.5)	74 (68.5)	
*Mid-lower back*			0.0001
Yes	11 (19.3)	58 (53.7)	
No	46 (80.7)	50 (46.3)	
*Upper abdomen*			<0.01
Yes	9 (15.8)	43 (40.0)	
No	48 (84.2)	65 (60.0)	
*Lower abdomen*			<0.001
Yes	14 (24.6)	58 (53.7)	
No	43 (75.4)	50 (46.3)	
*Upper arms*			0.42
Yes	2 (3.5)	7 (6.5)	
No	55 (96.5)	101 (93.5)	
*Lower arms*			<0.01
Yes	3 (5.3)	24 (22.2)	
No	54 (94.7)	84 (77.8)	
*Lower extremity*			<0.0001
Yes	9 (15.8)	61 (56.5)	
No	48 (84.2)	47 (43.5)	

* Five nursery workers did not provide responses to these questions.

### 3.2. Maternal, Birth and Infant Health Outcomes

*Maternal health.* There were no significant differences between nursery and fernery workers in report of maternal diagnosis of high blood pressure (*p* = 0.38), diabetes or high blood sugar (*p* = 0.28) or premature labor (*p* = 0.76) experienced during their most recent pregnancy. Women were also asked to report on health symptoms experienced during their last pregnancy. Compared to nursery workers, a larger proportion of women working in ferneries reported having eye irritations (17% *vs.* 3%) (*p* < 0.01) and throat irritations (15% *vs.* 3%) (*p* = 0.02) during their most recent pregnancy. Nursery workers reported significantly more nausea-related symptoms than fernery workers (46% *vs.* 31%) (*p* < 0.05) (Table 4).

*Birth outcomes*. We did not observe a statistically significant difference between occupational groups for pregnancy complications (*p* = 0.36), low birth weight delivery (*p* = 0.29) or preterm delivery (*p* = 0.88) (Table 5).

*Infant health*. There was no difference in self-report of infant health complications in the first year of life between mothers from the two agricultural groups (10.0% *vs.* 10.4%, *p* = 0.94). More than half of the women in both groups reported they breastfed their infants and continued to breastfeed to almost six months of age of their infants. Among fernery workers, 24.7% reported their child had been diagnosed by a healthcare provider with any (chronic) health problems, including respiratory or breathing problems and psychological or learning problems (*p* = 0.01), compared to only 8.5% of nursery workers. Women were also asked to provide details about the child’s diagnosed health problem. Results showed that significantly more mothers working in ferneries (24.7%) reported a child diagnosis of a chronic health problem compared to nursery workers (8.5%) (*p* = 0.01). Fernery workers were also 2.7-times more likely to report a child diagnosis of a respiratory condition compared to women working in nurseries (17.6% *vs.* 6.4%, *p* = 0.04) (Table 5).

**Table 4:** Maternal health outcomes for women working in nursery and fernery operations in Central Florida (*n* = 170), 2011.

Maternal health	Nursery * (N = 62) n (%)	Fernery * (N = 108) n (%)	*p-*Value
**Health Diagnosis during pregnancy**			
*High blood pressure*			0.38
Yes	4 (6.8)	6 (10.0)	
No	55 (93.3)	54 (90.0)	
*Diabetes or High Blood Sugar*			0.28
Yes	11 (18.6)	7 (11.7)	
No	48 (81.4)	53 (88.3)	
*Premature Labor*			0.76
Yes	9 (15.3)	8 (13.3)	
No	50 (84.7)	52 (86.7)	
**Health symptoms during pregnancy**			
*Nausea*			<0.05
Yes	29 (46.0)	33 (30.8)	
No	34 (54.0)	74 (69.2)	
*Eye irritations*			<0.01
Yes	2 (3.2)	18 (16.7)	
No	60 (96.8)	90 (83.3)	
*Throat irritations*			0.01
Yes	2 (3.2)	16 (14.8)	
No	60 (96.8)	92 (85.2)	
*Headache*			0.84
Yes	26 (41.9)	47 (43.5)	
No	36 (58.1)	61 (56.5)	
*Dizziness and Fainting*			0.17
Yes	14 (22.6)	35 (32.4)	
No	48 (77.4)	73 (67.6)	
*Blurred Vision*			0.93
Yes	10 (16.1)	18 (16.7)	
No	52 (83.9)	90 (83.3)	
*Swelling*			0.59
Yes	21 (33.9)	41 (38.0)	
No	41 (66.1)	67 (62.0)	
*Numbness and tingling of hands and legs*			0.81
Yes	13 (21.0)	21 (19.4)	
No	49 (79.0)	87 (80.6)	
*Skin Rash*			0.16
Yes	6 (9.7)	19 (17.6)	
No	56 (90.3)	89 (82.4)	
*Urinary Tract Infection*			0.55
Yes	13 (21.0)	27 (25.0)	
No	49 (79.0)	81 (75.0)	
*Fetal movement increased and decreased movement while at work*			0.14
Yes	9 (14.5)	8 (7.4)	
No	53 (85.5)	100 (92.6)	
*Coughing/wheezing*			0.41
Yes	4 (6.4)	11 (10.2)	
No	58 (93.6)	97 (89.8)	
*Heart racing*			0.77
Yes	6 (9.7)	12 (11.1)	
No	56 (90.3)	96 (88.9)	
*Spotting or vaginal bleeding*			0.33
Yes	5 (8.1)	14 (13.0)	
No	57 (91.9)	94 (87.0)	
*Constipation/diarrhea*			0.14
Yes	5 (8.1)	3 (2.8)	
No	57 (91.9)	105 (97.2)	
*Unsteady on feet/falls while working*			0.21
Yes	4 (6.5)	15 (13.9)	
No	58 (93.5)	93 (86.1)	

* Note: A total of three missing responses for nursery workers and 48 missing responses for fernery workers were observed for survey questions related to health diagnosis during pregnancy.

**Table 5:** Birth and infant health outcomes in first year of life reported by women working in nursery and fernery operations in Central Florida (*n* = 170), 2011.

Birth Outcomes	Nursery (N = 62) *n (%)*	Fernery (N = 108) *n (%)*	*p-*Value
*Pregnancy Complication*			0.36
Yes	60 (96.8)	101 (93.5)	
No	2 (3.2)	7 (6.4)	
*Preterm (<37 weeks)*			0.88
Yes	7 (11.3)	13 (12.0)	
No	55 (88.7)	95 (88.0)	
*Low birth weight (<2500 grams)*			0.29
Yes	5 (8.3)	4 (4.0)	
No	55 (91.7)	97 (96.0)	
Missing	2	7	
**Infant Outcomes**			
*Infant health complications ^a^*			0.94
Yes	6 (10.0)	11 (10.4)	
No	54 (90.0)	95 (89.6)	
Missing	2	2	
*Breastfeeding*			0.67
Yes	36(61.0)	65 (64.4)	
No	23 (39.0)	36 (36.6)	
Missing	3	7	
*Age of child in months when stopped breastfeeding (mean ± SD)*	5.9 ± 5.4	5.4 ± 5.2	0.62
*Child diagnosis of chronic health problem ^b^*			0.01
Yes	5 (8.5)	25 (24.7)	
No	54 (91.5)	76 (75.3)	
*Child diagnosis of respiratory condition*			0.04
Yes	4 (6.4)	19 (17.6)	
No	89 (93.6)	89 (82.4)	

^a^ Survey question: Did a healthcare provider tell you that the baby had any birth defects or other diseases from the time you delivered until one year of age?; ^b^ survey question: Has your child ever been diagnosed by a healthcare provider with any health problems, including respiratory or breathing problems, like asthma, psychological or learning problems, or any other chronic health problems?

*Haitian versus Mexican women.* Because 1/3 of nursery workers were Haitian immigrants, we decided to conduct a sub-analysis examining differences between the two agricultural industries based on nationality. Haitians told their supervisors almost two months later than their Mexican coworkers that they were pregnant (*p* < 0.001). Haitians also reported entering prenatal care at least eight weeks later than their Mexican counterparts (*p* < 0.001). In terms of work practices, Mexican workers were told to alter their work routine more often than Haitians (*p* < 0.001). However, Haitians were more likely to stop work due to a reported inability to make changes at work (*p* < 0.001). In terms of pesticide exposure symptoms, Mexican women reported more nausea/vomiting (*p* = 0.003), more dizziness/fainting (*p* < 0.001) and eye irritations (*p* = 0.04). Haitian women were more likely to report tachycardia/rapid heartbeat (*p* = 0.02). There were no differences between Mexican and Haitian mothers in pregnancy complications (*p =* 0.53), low birth weight (*p* = 0.25) or preterm deliveries (*p =* 0.17). Mexican mothers were more likely to self-report that their child was ever diagnosed with a chronic medical problem compared to Haitian mothers, who reported no chronic medical conditions among their children (*p* < 0.01).

## 4. Discussion

Previous research has shown that an average of half the workers in Central Florida ferneries and nurseries are women, and the majority of these women are of childbearing age [21]. Farmworkers are particularly at risk for the adverse effects of pesticide exposure, ergonomic stressors, heat strain and other occupational hazards during pregnancy. Despite labor-intensive conditions and other occupational risks, including long workdays in hot and humid environments, the findings from our study revealed that more than half of the women surveyed from each industry worked during their entire pregnancies. One interesting finding was that more than one third of fernery workers chose not to communicate with their supervisors about their pregnancies compared to only 11% of nursery workers. It may be that many of the women working in ferneries did not feel comfortable talking with their supervisors about possible modifications to their work tasks or simply that there are not that many ways to vary work tasks in fernery operations. However, most women working in ferneries followed the guidance of their healthcare providers and made changes to their regular work routine, including reducing work hours and taking more frequent breaks. Given the cross-sectional design of this study, we could not determine whether women who could modify their work tasks would have fewer musculoskeletal complaints or if having pain at work led to the modification of tasks. Environmental and occupational health assessment should be an essential component of the healthcare provider and farmworker patient interaction, but research shows that healthcare providers do not receive specialized education on the potential of occupational exposures to affect reproductive health of farmworker populations, especially as it pertains to adverse pregnancy outcomes [23].

Prenatal care plays a vital role in fostering healthy pregnancies and reducing adverse health outcomes for infants, including low birth rates [24]. Farmworkers face many barriers in accessing healthcare resources, including inadequate transportation, increased mobility from the migratory nature of work, low educational attainment, language and cultural differences, financial constraints, fear of the U.S. medical system and lack of health insurance and documentation [25,26,27,28,29]. It would seem that these barriers could be especially challenging for farmworker women and their infants who seek prenatal and postnatal care. Yet, our data showed that more than 97% of women from these two agricultural communities accessed prenatal care in the first trimester of pregnancy. Prior studies revealed that only 42% of migrant and seasonal farmworker women accessed prenatal care within the first three months of their pregnancy compared to national estimates reporting that three in four Mexican-American women access early prenatal care [30,31]. One reason for the higher rate of prenatal care among fernery and nursery workers may be that the state of Florida has presumptive Medicaid eligibility for pregnant women that provides temporary coverage based initially on limited intake information. This early initiation of prenatal care may contribute to the overall healthy pregnancy outcomes in this population of female farmworkers, especially to the lower than average proportion of low birth weight deliveries. Additionally, the community setting may have contributed to healthier pregnancy outcomes in this population of female farmworkers. The community organization working on this project believes that doctors in a rural neighboring community clinic with a strong FWAF presence were more likely to ask farmworkers in our study if they worked in agriculture.

The Centers for Disease Control and Prevention’s (CDC) Pregnancy Nutrition Surveillance System collects prenatal and postnatal data on women and children enrolled in publicly-funded assistance programs. Data from four states compared pregnant-related health behaviors for migrant (*n* = 4840) and non-migrant women (*n* = 610,728). The two groups were similar in terms of prevalence for low birth weight, preterm births and small-for-gestational-age infants. Data on migrant women showed that 6.7% of pregnancies resulted in low birth weight, and 9.9% of these women had a preterm birth [32]. While the percentage of low birth weight babies was lower among women in our study (5.3), the proportion of preterm pregnancies (11.8%) was slightly higher than CDC surveillance data. Hispanic immigrants born outside the U.S. have a lower risk for preterm low birth rate than non-Hispanic whites [33], though U.S.-born Hispanics have a similar or greater risk compared to non-Hispanic whites [34], which some suggest could be attributable in part to occupational hazards, including repetitive motion, physically demanding and uncomfortable body postures [32]. In our study, we observed no low birth weight deliveries and only one preterm delivery in Haitian women. Recent research focused specifically on Haitian immigrant women is very limited. One study of 31,663 Haitian-born women residing in Quebec shows that during a 25-year period (1981–2006), 8.5% had pre-term birth and 7.3% had low birth weight infants [33]. While results demonstrated significant, yet surprising differences in employer notification of pregnancy, entry into prenatal care, maternal health symptoms and the self-report of chronic health conditions in children between Haitian and Mexican farmworker women, there is no recent literature on the differences between these two ethnic groups. The importance of social and cultural factors that may influence workplace risk and pregnancy health between these two ethnic groups merits more study.

Pregnancy strains the musculoskeletal system and alters the mother’s center of gravity, resulting in an increased risk of musculoskeletal injury. Elevated risk of injury for pregnant women is further compounded by work-related ergonomic factors, including heavy workload, repeated bending/squatting, repetitive hand and body motion, standing for long periods of time, strenuous physical work including lifting and twisting and psychosocial stress. We found that fernery workers had more frequent pain complaints during pregnancy when compared to nursery workers, suggesting that work in fernery operations may be more physically demanding than nursery work. More than half of women working in ferneries experienced pain in their mid-lower back, lower abdomen and lower extremities, while one in five reported pain in their lower arms, which includes hands and wrists. Physically strenuous work conditions (e.g., heavy lifting, frequent bending) have long been recognized as an established risk factor for spontaneous abortion, low birth weight and preterm births [34]. In a review of 29 studies of women employed in non-agricultural occupations [17], occupational fatigue was defined as a scored criterion combination of prolonged standing, working with industrial machines, carrying of significant loads, repetitive physical tasks, exposure to increased noise, vibration or cold or working with chemicals. Occupational fatigue significantly increased the risk of preterm birth (RR 1.63, 95% CI: 1.33–1.98). Physically demanding work, defined as repetitive lifting or manual labor with exertion, was also associated with an increased risk of preterm birth and small size for gestation. In the Netherlands, women in the highest tertile of occupational physical activity during the first trimester were 1.58 (95% CI: 1.02–2.44) times as likely to deliver an infant in the lowest tertile for birth weight [35]. Similarly, an Indian cohort of female farmworkers reported an inverse relationship between daily physical activity and birth weight [19]. While we did not observe group differences in spontaneous abortion, preterm labor or birth weight in our sample of female farmworkers, more research examining musculoskeletal strain in pregnant farmworker women is needed to better understand the impact of a physically demanding job on pregnancy health and to identify interventional strategies to safeguard against work-related musculoskeletal injuries while pregnant.

Given that U.S. female farmworkers are predominantly Hispanic, the Hispanic epidemiologic paradox creates a challenge in determining if a true risk to reproductive health exists for farmworkers. Data trends in birth outcomes over the past two decades reveal that the longer Hispanic women live in the United States, the more likely they are to experience higher rates of preterm and low birth weight deliveries [36,37]. We did not obtain information in this study on the length of time the pregnant women had been in the United States, but the FWAF describes both the fernery and nursery worker communities as being non-migratory and more settled. Farm work is often the primary occupation for a majority of Hispanic families who migrate to the United States, suggesting that prolonged occupational exposure to work hazards may be an important contributing factor for poorer birth outcomes. Further, chronic health effects are highly prevalent in farmworker children, including developmental and cognitive delays and respiratory illnesses, [38,39,40,41,42,43] and may also provide additional evidence for the long-term and multigenerational effects of occupational and environmental exposures endured by agricultural workers.

While our results suggest minimal differences in pregnancy outcomes between women in these two agricultural industries, we observed a significantly higher proportion of fernery workers who reported that their child had been diagnosed with a chronic health problem in the first year of life. Among infants with a chronic health diagnosis, the majority of these self-reported diagnoses were respiratory-related conditions. Research has shown that a variety of factors can contribute to respiratory illnesses, including parents’ occupational exposures to pesticides, which can be brought into households on work clothing, and environmental factors, such as deteriorated housing conditions [44,45,46,47].

Workers in the two agricultural industries discussed here share similar cultural and socioeconomic characteristics, but we know that their work exposures can differ. We were unable to determine similarities or differences in the housing quality of our study participants. Nursery and fernery workers generally live in mobile homes, single-family residences, apartments or duplex/triplex units [48], as do most farmworkers in Florida. Housing conditions for both nursery and fernery workers are typical of housing conditions for farmworkers throughout the Southeast [49], with common issues of substandard conditions, overcrowding and defective or nonexistent appliances. Future studies of this population should include home environmental assessments along with occupational hazard assessment to more accurately determine factors associated with this increase in infant respiratory problems.

### Strengths and Limitations

One limitation in this retrospective, cross-sectional study is the potential for maternal recall bias resulting in significant over- or under-reporting of pregnancy and infant health outcomes (*i.e.*, outcome misclassification). Less than 10% of our sample reported on their most recent pregnancy occurring within one year of the survey completion date in the summer of 2011, leaving more than 90% reporting on their last pregnancy dating back several years. Research with Norwegian female farmers has shown improved recall of adverse birth outcomes, such as small for gestational age and late pregnancy loss, with a previous pregnancy loss; however, birth records proved more reliable among mothers without a previous pregnancy loss [50]. Additionally, interviewer and low-response bias are important study limitations to consider in cross-sectional studies. To reduce interviewer bias, all community interviewers (four interviewers in Apopka and three interviewers in Pierson) had at least a high school education and experience working with the FWAF on various issues important to farmworker health and safety and were trusted by community members. Each interviewer received 8 h of training over a two-day period on how to conduct the interviews. Moreover, participation rates were high—a total of three women approached refused participation—largely due to the strong FWAF presence in recruitment areas, further reducing the potential for self-selection bias in our study.

We used a snowball sampling approach to ensure adequate sampling and recruitment of female farmworkers in nurseries and ferneries, a vulnerable and hard-to-reach population. One limitation of this sampling approach is the likelihood of selection bias; however, a larger sample size, much like the one employed in our study, may help to reduce selection bias in studies using snowball sampling methodologies [45].

While the literature supports that work in agriculture is a risk factor for adverse birth outcomes, few studies have been able to capture the lived experience of women working in agriculture during pregnancy. One key advantage is that the current community-academic partnership was able to successfully obtain self-reported pregnancy health data for a hard-to-reach female farmworker population. The purpose of this paper was to compare the pregnancy histories of two types of agricultural workers, those employed in nursery operations and those in ferneries. We have previously described the work conditions and differences in these two types of agricultural settings [51]. We did not ask participants the name of their employers, since that could inhibit participation in the study; therefore, we can only describe differences between the two employment settings and not among different employers within the two employment settings. However, in our formative research with this population, we did not find evidence that the work conditions vary greatly from one employer to another within each setting [22]. Our future work will extend beyond these two employment settings and document the working conditions and pregnancy outcomes of women working in other types of agricultural industries, including citrus and vegetable crops. Future studies with this population might include a prospective birth cohort to quantify and compare multiple environmental exposures for pregnant women working in distinct types of agriculture. Using a birth cohort study design, researchers could also investigate the potential effects of these maternal environmental exposures pre- and post-natally on an array of early child health outcomes, including adverse pregnancy events, respiratory illness and neurodevelopmental performance.

## 5. Conclusions

This study provides insight into the pregnancy experience of a large number of farmworker women with access to prenatal care, but who experience work conditions that pose threats associated with chemical, musculoskeletal and heat exposures. In this exploratory analysis, we observed important differences in work-related behaviors and maternal and infant health outcomes for a sample of female farmworkers working while pregnant in two distinct agricultural industries. Although the results of this study must be interpreted with caution, the findings lend support to the hypothesis that parental occupation in certain types of agriculture, such as ferneries, is a potential risk factor for respiratory illness in their children. Results are not generalizable and might differ for farmworker women in other agricultural industries in which women are less likely to get early and/or adequate prenatal and/or consistent care, such as farmworker women that migrate with the crop/seasons. Additionally, the farmworker women in the current study all lived and worked in areas where the FWAF had a strong community presence.

## Supplementary Files

Supplementary File 1
