# Supplementary material for: Occupational Risks and Pregnancy and Infant Health Outcomes in Florida Farmworkers
Source: Int J Environ Res Public Health. 2014 Aug 6;11(8):7820–40. doi: 10.3390/ijerph110807820 (PMC4143835; doi:10.3390/ijerph110807820)
Supplement: Supplementary File 1 [file ijerph-11-07820-s001.pdf]

A. Participante #: ______________	B. Entrevistadora: __________________	C. Fecha: ___________________

PARA SER COMPLETADA POR LA ENTREVISTADORA:
D. Por favor indique la apariencia racial del entrevistado:

☐ 1 Negro
☐ 2 Blanco
☐ 3 Más de una raza
☐ 99 No sabe

Encuesta sobre el Embarazo en el Trabajo

Voy a hacerle algunas preguntas sobre su experiencia como mujer en edad reproductiva que trabaja en la hojita/ la nurseria. La encuesta nos debe tomar unos _____ minutos. Vamos a comenzar con algunas preguntas básica acerca de usted y de donde viene

SECCION A. DEMOGRAFICA

1. ¿Qué edad tiene? __ __ Años

2. ¿En qué país nació?

☐ 1 Estados Unidos
☐ 2 México
☐ 3 Haiti
☐ 4 Otro, E**SPECIFIQUE** ________________________

3. ¿En que condado esta actualmente viviendo? ________________________

4. ¿Cuál es su estado civil?

☐ 1 Casada
☐ 2 Union Libre/Viviendo con su pareja sin estar casados
☐ 3 Soltera
☐ 4 Divorciada
☐ 5 Viuda

5. ¿Qué idioma habla originalmente? ________________________

6. ¿Cuántos años de escuela terminó en su país de origen? ________________________

7. ¿Cuántos años de escuela terminó en los Estados Unidos? ________________________

8. ¿Se considera usted Hispana/Latina?

☐ 1 Si

☐ 2 No

☐ 77 SE NIEGA A CONTESTAR

☐ 99 NO SABE

9. ¿Está actualmente embarazada?

☐ 1 Si

☐ 2 No

☐ 77 SE NIEGA A CONTESTAR

☐ 99 NO SABE

SECCION B. PRACTICAS LABORALES

Las siguientes preguntas son sobre el tipo de trabajo que hace y las tareas que este trabajo implica.

10. ¿Esta actualmente empleada?

☐ 1 No

☐ 2 Si, tiempo parcial [menos de 35 horas a la semana]

☐ 3 Si, tiempo completo [mas de 35 horas a la semana]

☐ 77 SE NIEGA A CONTESTAR

☐ 99 NO SABE

11. En todo lo que va de su vida, ¿cuánto tiempo tiene trabajando en las norserias/con la hojita?____________________

12. Además de su trabajo en las norserias/con la hojita ¿ha hecho algun otro tipo de trabajo en la agricultura?

☐ 1 Si

☐ 2 No

☐ 77 SE NIEGA A CONTESTAR

☐ 99 NO SABE

13. En una semana típica, ¿cuál de las siguientes tareas hace en su trabajo?

	1 Frecuentemente	2 Algunas veces	3 Nunca
13A. Plantando (poniendo retoños en macetas - Poteando)	☐	☐	☐
13B. Cortando o podando	☐	☐	☐
13C. Moviendo plantas o arboles	☐	☐	☐
13D. Empacando y poniendo ordenes	☐	☐	☐
13E. Desempacando retoños	☐	☐	☐
13F. Lavando, limpiando plantas y/o arboles	☐	☐	☐
13G. Deshierbando (limpiando monte)	☐	☐	☐
13H. Clasificando plantas	☐	☐	☐
13I. Transportando	☐	☐	☐
13J. Cargando a mano	☐	☐	☐
13K. Mesclando/rociando/sprayando pesticidas u otros químicos	☐	☐	☐
13L. Limpiando el área de trabajo	☐	☐	☐
13M. Operando el montacargas (forklift) u otra maquinaria	☐	☐	☐
13N. Supervisando otros trabajadores	☐	☐	☐
13O. Otra cosa (Por favor especifique)			

14. En la última semana, cuando estuvo en el trabajo, cuanto timepo estuvo hacienda lo siguiente:

	1 Frecuentemente	2 Algunas veces	3 Nunca
14A. Sentada	☐	☐	☐
14B. Parada	☐	☐	☐
14C. Caminando	☐	☐	☐
14D. Cargando	☐	☐	☐
14E. Agachada	☐	☐	☐
14F. Cortando/Usando herramientas de mano	☐	☐	☐
14G. Arrodillada	☐	☐	☐
14H. En un lugar muy caliente o bajo el sol	☐	☐	☐
14I. Retorciéndose	☐	☐	☐
14J. Alcanzando cosas por encima de su cabeza	☐	☐	☐
14K. En cuclillas	☐	☐	☐

15. Durante el verano, con que frecuencia siente _______ (SINTOMA) mientras trabaja:

	1 Frecuentemente	2 Algunas veces	3 Nunca
15A. Fatiga	☐	☐	☐
15B. Debilidad	☐	☐	☐
15C. Perdida de coordinación	☐	☐	☐
15D. Mareos	☐	☐	☐
15E. Nausea	☐	☐	☐
15F. Calambres musculares repentinos	☐	☐	☐
15G. Agotamiento por calor (bochorno)	☐	☐	☐
15H. Desmayos	☐	☐	☐

SECTION C. HIGIENE EN EL TRABAJO

Las siguientes preguntas son sobre su acceso a facilidades sanitarias en su lugar de trabajo

16A. En su lugar de trabajo ¿Tiene un lugar donde pueda lavar sus manos?

☐ 1 Siempre

☐ 2 Algunas veces

☐ 3 Nunca

☐ 77 SE NIEGA A CONTESTAR

☐ 99 NO SABE

16B. **SI SIEMPRE O ALGUNAS VECES:** En su trabajo, donde se lava las manos ¿Cón que frecuencia hay jabón y toallas de papel?

☐ 1 Siempre

☐ 2 Algunas veces

☐ 3 Nunca

☐ 77 SE NIEGA A CONTESTAR

☐ 99 NO SABE

17. Cuando esta trabajando en la nurseria o con la hojita ¿Con que frecuencia se lava las manos antes de comer?

☐ 1 Siempre

☐ 2 Algunas veces

☐ 3 Nunca

☐ 77 SE NIEGA A CONTESTAR

☐ 99 NO SABE

18. En su lugar de trabajo ¿tiene agua potable (que este lo suficientemente limpia como para beber?

☐ 1 Siempre

☐ 2 Algunas veces

☐ 3 Nunca

☐ 77 SE NIEGA A CONTESTAR

☐ 99 NO SABE

19A. ¿Con que frecuencia su trabajo tiene vasos desechables o algo con que beber?

☐ 1 Siempre

☐ 2 Algunas veces

☐ 3 Nunca

☐ 77 SE NIEGA A CONTESTAR

☐ 99 NO SABE

19B. **SI SIEMPRE O ALGUNAS VECES:** Si no le dan vasos desechables, con que frecuencia usa cualquier recipiente disponible para beber agua?

☐ 1 Siempre

☐ 2 Algunas veces

☐ 3 Nunca

☐ 77 SE NIEGA A CONTESTAR

☐ 99 NO SABE

20A. En su lugar de trabajo, ¿puede tomar agua a la hora que usted quiera?

☐ 1 Siempre

☐ 2 Algunas veces

☐ 3 Nunca

☐ 77 SE NIEGA A CONTESTAR

☐ 99 NO SABE

SI ALGUNAS VECES O NUNCA: Cuando no puede tomar agua a la hora que usted quisiera ¿Con que frecuencia se debe a una de las siguientes razones?

	1 Frecuentemente	2 Algunas veces	3 Nunca
20B. Estoy preocupada que mi supervisor me este observando y se enoje conmigo	☐	☐	☐
20C. No quiero tomar tiempo de mi trabajo	☐	☐	☐
20D. No hay agua de beber	☐	☐	☐

21A. ¿Normalmente hay baños en su lugar de trabajo?

☐ 1 Si, hay baño

☐ 2 Si, hay baños moviles/letrinas (portable toilets)

☐ 3 No

21B. SI **NO**: ¿A donde va a hacer del baño?

____

____

____

☐ 77 SE NIEGA A CONTESTAR

☐ 99 NO SABE

22A. En su lugar de trabajo ¿Puede ir al baño en el momento que usted quiere?

☐ 1 Siempre

☐ 2 Algunas veces

☐ 3 Nunca

☐ 77 SE NIEGA A CONTESTAR

☐ 99 NO SABE

SI ALGUNAS VECES O NUNCA: Cuando no puede ir al baño a la hora que usted quisiera ¿Con que frecuencia se debe a una de las siguientes razones?

	1 Frecuentemente	2 Algunas veces	3 Nunca
22B. Estoy preocupada que mi supervisor me este observando y se enoje conmigo	☐	☐	☐
22C No quiero tomar tiempo de mi trabajo	☐	☐	☐
22D. Los baños no estan limpios o les falta mantenimiento	☐	☐	☐

23. En su lugar de trabajo ¿le dan equipo de protección (por ejemplo: guantes, overoles, mascarillas, lentes)?

☐ 1 Siempre (PASE A LA PREGUNTA 25)

☐ 2 Algunas veces

☐ 3 Nunca

☐ 77 SE NIEGA A CONTESTAR

☐ 99 NO SABE

24. ¿Usted tiene que traer su propio equipo de protección (por ejemplo: guantes, overoles, mascarillas, lentes)?

- ☐ 1 Siempre
- ☐ 2 Algunas veces
- ☐ 3 Nunca
- ☐ 77 SE NIEGA A CONTESTAR
- ☐ 99 NO SABE

25. ¿En que momento se cambia de ropa después de haber salido del trabajo?

- ☐ 1 Inmediatamente
- ☐ 2 En los primeros 30 minutos de salir
- ☐ 3 Mas tarde de 30 minutos de haber salido
- ☐ 77 SE NIEGA A CONTESTAR
- ☐ 99 NO SABE

26. . ¿En que momento se baña cuando llega a casa después del trabajo?

- ☐ 1 Inmediatamente
- ☐ 2 En los primeros 30 minutos de salir
- ☐ 3 Mas tarde de 30 minutos de haber salido
- ☐ 77 SE NIEGA A CONTESTAR
- ☐ 99 NO SABE

SECCION D. CREENCIAS DE SALUD

Las siguientes preguntas son sobre sus sentimientos y creencias de los pesticidas

27. ¿Con que frecuencia piensa que tiene contacto con los pesticidas mientras trabaja en la nurseria/con la hojita?

- ☐ 1 Todos los días
- ☐ 2 Ocasionalmente
- ☐ 3 Nunca
- ☐ 77 SE NIEGA A CONTESTAR
- ☐ 99 NO SABE

28. En el mes pasado ¿Con que frecuencia le preocuparon los efectos de los pesticidas a su salud?

- ☐ 1 Todos los días
- ☐ 2 Ocasionalmente
- ☐ 3 Nunca
- ☐ 77 SE NIEGA A CONTESTAR
- ☐ 99 NO SABE

29. ¿Usted cree que alguna vez ha estado enferma por haber trabajado en lugares donde se han usado pesticidas?	☐ 1 Si	☐ 2 No	☐ 3 No estoy segura	☐ 77 Se niega a contestar
30. ¿Usted cree que los pesticidas puedan afectar a las personas que trabajan en las norserias o en la hojita?	☐ 1 Si	☐ 2 No	☐ 3 No estoy segura	☐ 77 Se niega a contestar
31. ¿Usted cree que los pesticidas puedan afectar la salud de los niños en los vientres de las madres que trabajan en la nurseria/ o en la hojita?	☐ 1 Si	☐ 2 No	☐ 3 No estoy segura	☐ 77 Se niega a contestar
32. ¿Usted cree que los pesticidas puedan afectar la salud de los niños que vivien con padres que trabajan en la nurseria/ o en la hojita?	☐ 1 Si	☐ 2 No	☐ 3 No estoy segura	☐ 77 Se niega a contestar
33. ¿Usted cree que tendra problemas de salud en futuro a causa de los pesticidas?	☐ 1 Si	☐ 2 No	☐ 3 No estoy segura	☐ 77 Se niega a contestar
34. ¿Usted cree que sus hijos esten sufriendo o sufriran de problemas de salud a causa de los pesticidas?	☐ 1 Si	☐ 2 No	☐ 3 No estoy segura	☐ 77 Se niega a contestar

SECTION E. HISTORIA REPRODUCTIVA

Ahora me gustaría preguntarle sobre las veces en las que ha estado embarazada.

35. ¿Cuántas veces ha estado embarazada? Por favor incluya a todos los niños vivos, abortos voluntarios e involuntarios (miscarriages), niños nacidos muertos, embarazos ectópicos, embarazos en los tubos de la matriz, y embarazos molares __ __ **embarazo(s) [00 = Ninguno]**

Ahora me gustaría preguntarle sobre cada uno de sus embarazos comenzando con su último embarazo.

36.	Dígame ¿Cuál fue el resultado de su embarazo **mas reciente**?<br>1. El niño nació vivo<br>2. Miscarriage (perdida del embarazo en las primeras 20 semanas de gestación)<br>3. Aborto<br>4. El niño nació muerto (nacido después de 20 semanas de gestación)<br>5. Embarazo Ectópico (embarazo en los tubos de la matriz)<br>6. Embarazo Molar<br>77. SE NIEGA A CONTESTAR<br>99. NO SABE	
37.	¿Cuántas semanas duro el embarazo?<br>**[USE MESES SI NO SABE CUANTAS SEMANAS]**<br>**[SI NO SABE, PREGUNTE SI]:** ¿Fue un embarazo a término? 37-40 semanas, o 9 meses?<br>**[94=NO FUE A TERMINO]**<br>**[95= A TERMINO]**<br>**[99 = NO SABE]**	__ __<br>**MARQUE: SEM/MES**
38A.	¿Estuvo trabajando durante este embarazo?<br>1 – SI, TRABAJO DURANTE TODO EL EMABRAZO **(PASE A LA PREGUNTA 39)**<br>2 – SI, PERO DEJO DE TRABAJAR ANTES QUE TERMINARA EL EMBARAZO<br>3 – NO, NO TRABAJO EN NINGUN MOMENTO DE ESTE EMBARAZO **(PASE A LA PREGUNTA 41)**<br>77 – SE NIEGA A CONTESTAR<br>99 – NO SABE	

38B.	SI no trabajo durante todo el embarazo. ¿En que semana dejo de trabajar? **[USE MESES SI NO SABE CUANTAS SEMANAS]**	__ __<br>**MARQUE: SEM/MES**
39.	¿Trabajo en la agricultura durante este embarazo?<br>1 – SI, EN UNA NURSERIA<br>2 – SI, CON LA HOJITA<br>3 – SI, EN AMBOS UNA NURSERIA Y LA HOJITA<br>4 – SI, EN OTRO TRABAJO AGRICOLA<br>5 – NO, NO TRABAJE EN LA AGRICULTURA<br>77 – SE NIEGA A CONTESTAR<br>99 – NO SABE	__<br>37B. Si 4, ¿que tipo?<br>___________________________________<br>___________________________________<br>___________________________________
40A.	¿Le dijo a su supervisor que estaba embarazada?<br>1 – SI<br>2 – NUNCA LE DIJE PERO SE DIO CUENTA<br>3 – NO **(PASE A LA PREGUNTA 41)**<br>4 –NO TRABAJE DURANTE ESTE EMBARAZO **(PASE A LA PREGUNTA 41)**<br>77 – SE NIEGA A CONTESTAR<br>99 – NO SABE	
40B.	**SI LE DIJO:** ¿En que momento le dijo que estaba embarazada?<br>**[USE MESES SI NO SABE CUANTAS SEMANAS]**<br>**[99 = NO SABE]**	__ __<br>**MARQUE: SEM/MES**
41.	¿Fumo durante este embarazo?<br>1 – SI<br>2 – ALGUNAS VECES<br>3 – NO<br>77 – SE NIEGA A CONTESTAR<br>99 – NO SABE	__

42.	¿Tomo alcohol durante este embarazo?<br>1 – SI<br>2 – ALGUNAS VECES<br>3 – NO<br>77 – SE NIEGA A CONTESTAR<br>99 – NO SABE	__
43.	¿Recibió cuidado prenatal durante este embarazo?<br>1 – SI<br>2 – NO<br>77 – SE NIEGA A CONTESTAR<br>99 – NO SABE	__
44.	¿Qué tipo de problemas tuvo para recibir atención prenatal durante este embarazo?<br>**MARQUETODAS LAS QUE APLIQUEN**	A. No consiguió hacer cita<br>B. No tenia dinero para recibir atención<br>C. Sin transportación<br>D. No tenía seguro médico<br>E. Sin Medicaid<br>F. Sin ayuda para cuidar a los otros hijos<br>G. Muy ocupada o con muchas otras preocupaciones para buscar cuidado prenatal<br>H. Tuvo que ocultar el embarazo<br>I. No existía atención médica en el area<br>J. Temerosa de perder días de trabajo y/o las consecuencias en su trabajo<br>K. Otra razón<br>Por favor especifique :<br>L. ____________________________<br>M. ___________________________<br>N. ____________________________

45.	¿En que semana de su embarazo recibió su primera atención prenatal?<br>**[USE MESES SI NO SABE CUANTAS SEMANAS]**<br>**[99 = NO SABE]**<br>**NO MARQUE NADA SI NO RECIBIO CUIDADO PRENATAL**	__ __<br>**MARQUE: SEM/MES**
46.	Durante su embarazo ¿Se sintió cómoda hablando con su medico y haciéndole preguntas?<br>1 – MUY COMODA<br>2 – MAS O MENOS COMODA<br>3 – SE SINTIO INCOMODA<br>77 – SE NIEGA A CONTESTAR<br>99 – NO SABE	__
47.	Durante su embarazo, ¿Su médico le pregunto sobre el tipo de trabajo que estaba haciendo?<br>1 – SI<br>2 – NO **[PASE A LA PREGUNTA 51]**<br>77 – SE NIEGA A CONTESTAR<br>99 – NO SABE	__
48.	¿Recibió indicaciones de su médico u enfermera para que cambiara algo en el trabajo que hacia por el tiempo en que duraba este embarazo?<br>1 – SI<br>2 – NO **(PASE A LA PREGUNTA 51)**<br>77 – SE NIEGA A CONTESTAR<br>99 – NO SABE	__
49<br>A-F.	**SI ASI FUE:** ¿Qué le pidió que cambiara?<br>**[MARQUE A LA DERECHA]**	A. Trabajar menos horas<br>B. Tomar descansos con mayor frecuencia<br>C. Estar más tiempo sentada que parada<br>D. Otro tipo de recomendación, por favor especifique:<br>E. ___________________________<br>F. ___________________________

49G.	Si le hicieron una recomendación ¿Pudo hacer los cambios en el trabajo para seguir trabajando?<br>1 – SI<br>2 – NO, TUVE QUE DEJAR DE TRABAJAR<br>3 – NO, SEGUI TRABAJANDO DE LA MISMA FORMA, CONTRARIO A LAS INDICACIONES DEL MEDICO/ENFERMERA<br>77 – SE NIEGA A CONTESTAR<br>99 – NO SABE	__
50.	Durante el embarazo ¿se le diagnosticó con:<br>**[PREGUNTE POR CADA DIAGNOSTICO]**<br>1 – SI<br>2 – NO<br>77 – SE NIEGA A CONTESTAR<br>99 – NO SABE	
50A.	Alta presión arterial, pre eclampsia, toxemia, o eclampsia?	__
50B.	Diabetes o alto nivel de azúcar en la sangre?	__
50C.	Parto prematuro o contracciones prematuras?	__
50D.	Alguna otra complicación en su embarazo? (Especifique por favor)	50E.____________<br>50F.____________<br>50G.____________

51.	Durante este último embarazo ¿tuvo alguno de los siguientes problemas?<br><br>**(LEA Y MARQUE LA LISTA A LA DERECHA]**<br><br>**MARQUE. N/A SI NINGUNO DE LOS PROBLEMAS FUERON REPORTADOS**	A. Dolor de cabeza<br>B. Nausea/vomito<br>C. Mareos/Desmayos<br>D. Vista Borrosa/Nublada<br>E. Hinchazón<br>F. Se le entumieron las manos o las piernas<br>G. Ronchas/Salpullido<br>H. Ojos irritados<br>I. Infección en las vías urinarias<br>J. Incremento o decremento de los movimientos del niño a la hora del trabajo<br>K. Mucha tos/dificultad para respirar<br>L. Taquicardia/El corazón muy acelerado<br>M. Garganta irritada<br>N. Sangrado vaginal o pantis manchadas<br>O. Constipación o diarrea<br>P. Piernas temblorosas/caídas mientras trabajaba<br>Q. N/A
52.	**[Si el participante no trabajo en la agricultura en este ultimo embarazo PASE A LA PREGUNTA 55]**<br><br>Nos podría mostrar donde tuvo dolor **mientras trabajaba** en la nurseria o la hojita estando embarazada.<br><br>**[COMPLETE BODY PAIN CHART Y MARQUE EN ESTA PAGINA AL TERMINAR]**	___ Body chart completed<br><br>**MARQUE N/A si no aplica**
53.	Nos podría mostrar donde tuvo ronchas/salpullido **cuando trabajaba** en la nurseria o la hojita estando embarazada.<br><br>**[COMPLETE RASH CHART Y MARQUE EN ESTA PAGINA AL TERMINAR]**	___ Rash chart completed<br><br>**MARQUE N/A si no aplica**
54.	¿En que fecha nació su hijo(a) o termino este embarazo)?<br><br>**[00/00/13]= ACTUALMENTE EMBARAZADA**<br><br>**[99/99/13]= NO SE ACUERDA**	__ __/__ __/__ __<br><br>MES/DIA/ANO

55.	¿En algún momento desde su nacimiento hasta el primer año de vida, el medico le dijo que el bebe tenía algún defecto de nacimiento o alguna otra enfermedad? (Pregunto incluso si el niño falleció)<br><br>1 – SI<br><br>2 – NO<br><br>77 – SE NIEGA A CONTESTAR<br><br>99 – NO SABE<br><br>**[SI ACTUALMENTE EMBARAZADA O NO RESULTO EN INFANTE VIVO PASE A LA PREGUNTA 63]**	SI contesta afirmativamente, por favor especifique:<br><br>__<br><br>**Si fue causa de muerte marque (D)**<br><br>55B.________________________<br><br>55C.________________________<br><br>55D.________________________<br><br>55E.________________________
56.	**[CONTINUE SOLO SI EL EMBARAZO TERMINO EN HIJO VIVO]**<br><br>¿Este ultimo hijo fue un niño o una niña?<br><br>NIÑO – 1<br><br>NIÑA– 2	__
57.	¿Cuál es el nombre de su niño(a)?	________________
58.	¿Qué peso tenia __________________ **[NOMBRE]** cuando nacio?<br><br>**[SI NO SABE]** ¿Sabe si _______________ **[NOMBRE]** pesando menos de 5 lbs 8 oz? (2500g)<br><br>MARQUE si 'NO SABE':<br><br>**9994:** < 5 lbs, 8 oz o 2500 gramos<br><br>**9995:** > 5 lbs, 8oz o 2500 gramos	__ lbs __ oz<br><br>Or<br><br>_______ kg

59.	¿Le dio el pecho a ____________________ **[NOMBRE]**?<br>1 – SI<br>2 – NO **(PASE A LA PREGUNTA 61)**<br>77 – SE NIEGA A CONTESTAR<br>99 – NO SABE	__
60.	¿Cuántos meses tenia _____________ **[NOMBRE]** cuando dejo de darle pecho?<br>**[94 = ACTUALMENTE AMAMANTANDO]**<br>**[95 = MENOS DE UN MES]**<br>**[77 = SE NIEGA A CONTESTAR]**<br>**[99 = NO SABE]**	__ __<br>MESES
61.	¿Qué edad tenía ___________ **[NOMBRE]** cuando usted regreso a trabajar?	_______________**MARQUE: SEM/MES/AÑOS**
62.	¿Alguna vez han diagnosticado a _________________ **[NOMBRE]** de alguna enfermedad incluyendo problemas respiratorios, asma, problemas psicológicos o de aprendizaje o cualquier otro problema crónico?<br>SI – 1 (ESPECIFIQUE)<br>NO – 2<br>SE NIEGA A CONTESTAR – 7<br>NO SABE – 9	__<br>SI contesta afirmativamente, por favor especifique:<br>A._______________<br>B._______________<br>C._______________

HÁBITOS PATERNALES		
63.	¿Vivió con el papa del niño mientras duro su embarazo?<br>1 – SI<br>2 – NO<br>77 – SE NIEGA A CONTESTAR<br>99 – NO SABE	__
64.	¿Durante este embarazo el padre de su hijo	
64A.	Trabajo en las norserias o en la hojita?<br>1 – SI<br>2 – NO<br>77 – SE NIEGA A CONTESTAR 99 – NO SABE	__
64B.	Aplico pesticidas, espray en el trabajo (sprayo)?<br>1 – SI<br>2 – NO<br>77 – SE NIEGA A CONTESTAR 99 – NO SABE	__

En esta última sección voy a preguntarle por el resto de sus embarazos comenzando con el más reciente después del que le acabo de preguntar.

(MENCIONE EL NOMBRE DEL ÚLTIMO HIJO SI ES POSIBLE)

	EMBARAZO	2nd más reciente	3rd más reciente	4th más reciente	5th más reciente	6th más reciente
65.	Díganos ¿Cual fue el resultado de sus anteriores embarazos comenzando con el penúltimo?<br>1. Niño(a) viva<br>2. Un aborto involuntario **con menos** de 20 semanas o 5 meses de embarazo (Miscarriage) .<br>3. Aborto<br>4. Nacido muerto (≥ 20 semanas o 5 meses de embarazo)<br>5. Embarazo Ectopic (tubal)<br>6. Embarazo Molar<br>77 – SE NIEGA A CONTESTAR<br>99. NO SABE	__	__	__	__	__
66.	¿Trabajo en la agricultura durante este embarazo?<br>1 – SI, EN UNA NURSERIA<br>2 – SI, CON LA HOJITA<br>3 – SI, EN AMBOS: NURSERIA Y LA HOJITA<br>4 – SI, EN OTRO TRABAJO AGRICOLA<br>5 – NO, NO TRABAJE EN LA AGRICULTURA<br>77 – SE NIEGA A CONTESTAR<br>99 – NO SABE	__<br>66B. Si 4, pregunte ¿que tipo de trabajo?<br>________________<br>________________	__<br>66B. Si 4, pregunte ¿que tipo de trabajo?<br>________________<br>________________	__<br>66B. Si 4, pregunte ¿que tipo de trabajo?<br>________________<br>________________	__<br>66B. Si 4, pregunte ¿que tipo de trabajo?<br>________________<br>________________	__<br>66B. Si 4, pregunte ¿que tipo de trabajo?<br>________________<br>________________
67.	¿Recibió cuidado prenatal durante este embarazo?<br>1 – SI<br>2 – NO **(PASE A LA PREGUNTA 70)**<br>77 – SE NIEGA A CONTESTAR<br>99 – NO SABE	__	__	__	__	__

	EMBARAZO	2nd más reciente	3rd más reciente	4th más reciente	5th más reciente	6th más reciente
68.	Durante el embarazo ¿se le diagnosticó con **[PREGUNTE POR CADA DIAGNOSTICO]?**<br>1 – SI<br>2 – NO<br>77 – SE NIEGA A CONTESTAR<br>99 – NO SABE					
69A.	Alta presión arterial, pre eclampsia, toxemia, o eclampsia?	__	__	__	__	__
69B.	Diabetes o alto nivel de azúcar en la sangre?	__	__	__	__	__
69C.	Parto premature o contracciones prematuras?	__	__	__	__	__
70.	¿En algún momento desde su nacimiento hasta el primer año de vida, el medico le dijo que el bebe tenía algún defecto de nacimiento o alguna otra enfermedad?<br>1 – SI **[Pregunte si fue mas de una enfermedad o defecto, y si fue causa de muerte]**<br>2 – NO<br>77 – SE NIEGA A CONTESTAR<br>99 – NO SABE	__<br>SI contesta afirmativamente, por favor especifique:<br>**Si fue causa de muerte marque (D)**<br>70B.____________<br>70C.____________<br>70D.____________<br>70E.____________	__<br>SI contesta afirmativamente, por favor especifique:<br>**Si fue causa de muerte marque (D)**<br>70B.____________<br>70C.____________<br>70D.____________<br>70E.____________	__<br>SI contesta afirmativamente, por favor especifique:<br>**Si fue causa de muerte marque (D)**<br>70B.____________<br>70C.____________<br>70D.____________<br>70E.____________	__<br>SI contesta afirmativamente, por favor especifique:<br>**Si fue causa de muerte marque (D)**<br>70B.____________<br>70C.____________<br>70D.____________<br>70E.____________	__<br>SI contesta afirmativamente, por favor especifique:<br>**If cause of death indicate - (D)**<br>70B.____________<br>70C.____________<br>70D.____________<br>70E.____________
71.	¿Qué peso tenia el/la niño(a) cuando nació?<br>**[SI NO SABE PREGUNTE SI FUERON MENOS DE 2 KILOS Y MEDIO -2500 GRAMOS o DE 5 LBS 8 OZ O]**<br>**9994:** < 5 lbs, 8 oz o 2500 gramos<br>**9995:** > 5 lbs, 8oz o 2500 gramos	__ lbs __ oz<br>Or<br>________ kg<br>MARQUE si 'NO SABE':	__ lbs __ oz<br>Or<br>________ kg	__ lbs __ oz<br>Or<br>________ kg	__ lbs __ oz<br>Or<br>________ kg	__ lbs __ oz<br>Or<br>________ kg

GRACIAS POR SU PARTICIPACION
